# Sub aortic tendon induced ST segment elevation – a new echo electrocardiographic phenomenon?

**DOI:** 10.1186/1476-7120-7-13

**Published:** 2009-03-24

**Authors:** James Ker

**Affiliations:** 1 Department of Physiology, University of Pretoria, PO Box 24318, Gesina, Pretoria, 0031, South Africa

## Abstract

The causes for ST-segment elevation other than myocardial infarction are numerous.

The existence of left ventricular false tendons has been known for more than a century. Currently, the clinical entities associated with these left ventricular false tendons include innocent murmurs and premature ventricular contractions.

A case report is presented where such a false tendon, attached to the interventricular septum, is responsible for striking ST-segment elevation in the anterior precordial leads.

It is proposed that this is a newly observed entity – that of subaortic tendon-induced ST-segment elevation. This is proposed as a totally benign phenomenon with the clinical importance in that it should not be confused with other pathological processes, such as the Brugada syndrome.

## Introduction

The first description of so called false tendons inside the left ventricle was published 115 years ago [1]. These structures are also described in the literature as: left ventricular moderator bands, anomalous cords, left ventricular bands and aberrant tendons [2]. Abdulla *et al *[3] examined these tendons histologically and suggested that they are intracavitary radiations of the bundle of His. Embryologically, these false tendons are thought to derive from the inner muscle layers of the primitive heart and in addition to Purkinje cells, they also contain myocardial fibers, blood vessels, connective tissue and fibrous tissue [2]. Anatomically, these tendons have been divided into longitudinal and transverse tendons – longitudinal tendons extending from the ventricular septum to the posteroapical wall and transverse tendons extending between the septum and the lateral wall [4]. These tendons have been shown to be a cause of functional ejection murmurs [2-5]. It has also been documented that they are associated with both uni-and multifocal premature ventricular contractions (PVC's) [4,6]. These PVC's are poorly controlled by antiarrhythmic drugs, but easily suppressed by exercise [4].

There are currently two hypotheses for the generation of these PVC's [4]: These tendons contain Purkinje fibers and it is known that the automaticity of Purkinje cells is increased by mechanical stretching [4,7]. It may be that mechanical stretching of the tendon can generate the PVC or alternatively, the mechanical strectch of the ventricular wall, where the tendon inserts, may trigger the PVC.

## Case report

A case report is presented where it is postulated that a left ventricular false tendon, attaching to the interventricular septum in a subaortic location, is responsible for striking ST-segment elevation in the anterior precordial leads.

**Figure 1:**
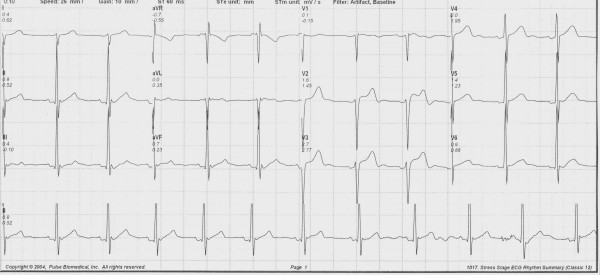
**File format: JPEG**. Title: 12-lead electrocardiogram. Description: This is the 12-lead electrocardiogram which clearly demonstrates the striking ST-segment elevation in leads V2 and V3.

A 34-year old Caucasian male was referred for a cardiovascular examination, because of an abnormal electrocardiogram, demonstrating striking ST-segment elevation in leads V1, 2 and 3 (Figure 1). The patient is totally asymptomatic. The ECG was done by his primary care physician for insurance purposes for an insurance policy he applied for. He is an athlete who competes in marathon running in his spare time.

The clinical examination was normal. No chest pain or any symptoms were present. Troponin T and creatine kinase levels were all normal. Other known causes for ST-segment elevation, such as hypothermia, trauma, hypercalcemia and hyperkalemia were all excluded. Echocardiography demonstrated a heart without any pathological findings.

The only peculiarity noted was the presence of a left ventricular false tendon, extending between the apical wall and the interventricular septum (see Additional file 1 and Additional file 2). Note the parasternal, long-axis view from a transthoracic echocardiographic image, demonstrating the false tendon, marked with + (See Additional file 1, Additional file 2 and Additional file 3.) The subaortic location of implantation is clearly shown. Also note the same false tendon from the parasternal short axis view, marked with + (see Additional file 4). Note the localized, hypertrophic response at the site of implantation (see Additional file 4, Additional file 5 and Additional file 6).

The patient was closely followed for a period of three years. During this period no symptoms or signs of any cardiovascular disease and specifically no rhythm disturbances was ever present. He remains totally asymptomatic to date.

## Discussion

The differential diagnosis of ST-segment elevation is wide and diverse, and includes the following [8]: myocardial ischemia or infarction, Prinzmetal angina pattern, Takotsubo cardiomyopathy, ventricular aneurysm, pericarditis, normal variant ("early repolarization"), left ventricular hypertrophy, left bundle branch block, other causes of myocardial injury, such as myocarditis, trauma or tumor invading the left ventricle, hypothermia, after DC cardioversion, hyperkalemia, hypercalcemia, type 1C antiarrhythmic drugs, intracranial hemorrhage and the Brugada pattern.

Three aspects are important in the discussion of this case:

Firstly, Loukas et al [9] divided false tendons into five categories, according to the site of implantation. Ker [10] described a sixth type of false tendon where the specific location is between the subaortic portion of the interventricular septum and the left ventricular free wall. Six cases were described and in all of them a striking, localized ventricular, hypertrophic response was present at the site of subaortic implantation. This condition was named "false tendon-induced subaortic hypertrophy" and it was proposed as a new variant of hypertrophic cardiomyopathy [10]. This case clearly belong to this sixth group of false tendons with a clear subaortic implantation location.

Secondly, repolarization changes in leads V1–V3 can be associated with Brugada syndrome. This case does not fulfill the criteria for Brugada syndrome [11]: Three ECG patterns for Brugada syndrome have been identified but only a "coved" or type I ECG segment elevation is presently considered diagnostic for the disease [11]. The other crucial aspect in the diagnosis of Brugada syndrome is the criterion for structurally normal hearts [11]. This case clearly does not demonstrate a structurally normal heart, as it demonstrates the false tendon-induced subaortic hypertrophy variant of hypertrophic cardiomyopathy. In order to accept a type II ECG ("saddle-back" elevation) as Brugada syndrome one needs genetic confirmation with a structurally normal heart [11].

Thirdly, hypertrophy is a mechanism (or response) of the heart to reduce stress on the ventricular wall [10]. It has been shown that mechanical stress on the ventricular wall can elicit a wide variety of auto-and paracrine responses, leading to the local secretion of a wide variety of growth factors, such as endothelin 1, angiotensin II and insulin like growth factor [10]. Therefore, scientifically it is clearly conceivable that a false tendon inserting itself in a subaortic location, will cause a localized stretch response at the implantation site and leading in some instances to a localized, hypertrophic response, as shown before [10]. It is therefore quite plausible that the localized, septal hypertrophic response is the reason for the observed repolarization changes.

In conclusion, a case report is presented of a young, healthy man with a "saddle back" ST-elevation of the precordial leads. The cause for this phenomenon is the sixth type of false tendon, the subaortic false tendon [10] leading to a localized hypertrophic response. It is proposed that this is a totally benign phenomenon and that care should be taken in the young, healthy patient not to confuse this entity with serious disorders, like the Brugada syndrome.

## Consent

Written informed consent was obtained from the patient for publication of this case report and accompanying images. A copy of the written consent is available for review by the Editor-in-Chief of this journal.

## Competing interests

The author declares that they have no competing interests.

## Supplementary Material

Additional file 1 **Parasternal long axis view of muscular tendon.** This is a movie clip of the parasternal long axis view of the sub-aortic muscular tendon, attached to the interventricular septum. Click here for file

Additional file 2 **Apical origin.** This image shows the apical origin of the muscular tendon. Click here for file

Additional file 3 **Muscular nature.** Image clearly demonstrates the muscular nature of the tendon. Click here for file

Additional file 4 **Short axis view.** Short axis image. Note the localized hypertrophic response at the septal area of implantation. Click here for file

Additional file 5 **Localized hypertrophy.** Movie clip demonstrating the localized, hypertrophic response at the site of septal implantation. Click here for file

Additional file 6 **Septal implantation.** Image clearly showing the septal implantation of the muscular tendon. Click here for file
