# Supplementary figures and images for: Sub aortic tendon induced ST segment elevation – a new echo electrocardiographic phenomenon?
Source: Cardiovasc Ultrasound. 2009 Mar 24;7:13. doi: 10.1186/1476-7120-7-13 (PMC2666638; doi:10.1186/1476-7120-7-13)

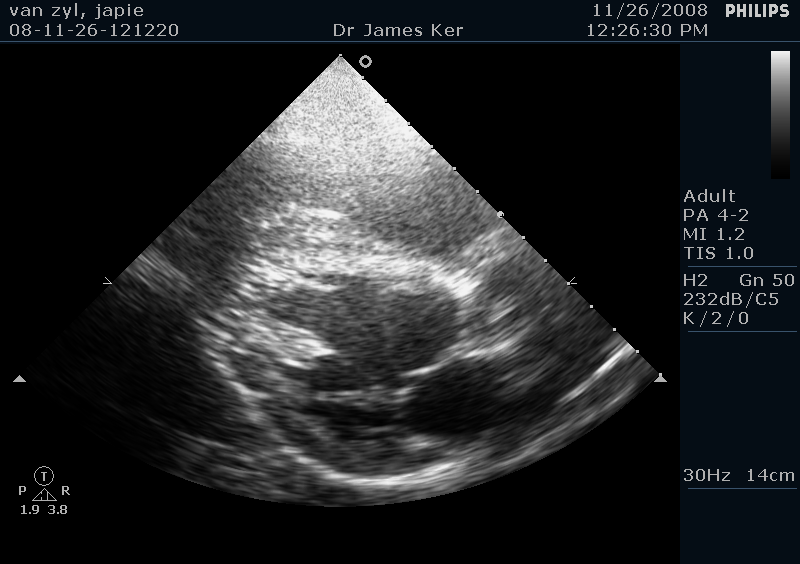

Supplement: Additional file 2 — Apical origin. This image shows the apical origin of the muscular tendon. [file 1476-7120-7-13-S2.bmp]

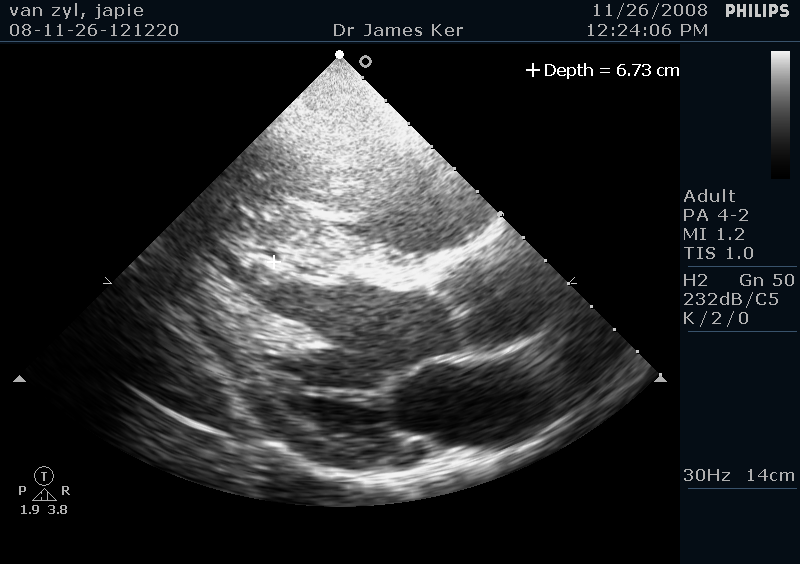

Supplement: Additional file 3 — Muscular nature. Image clearly demonstrates the muscular nature of the tendon. [file 1476-7120-7-13-S3.bmp]

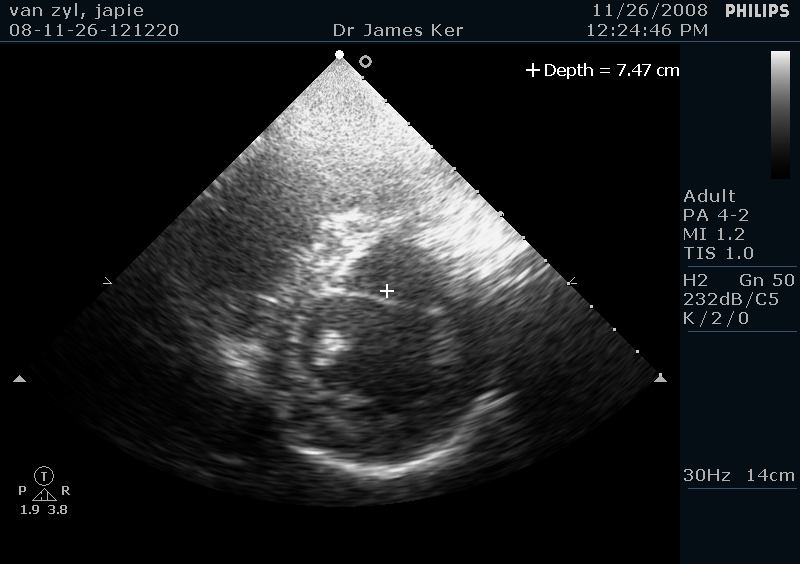

Supplement: Additional file 4 — Short axis view. Short axis image. Note the localized hypertrophic response at the septal area of implantation. [file 1476-7120-7-13-S4.bmp]

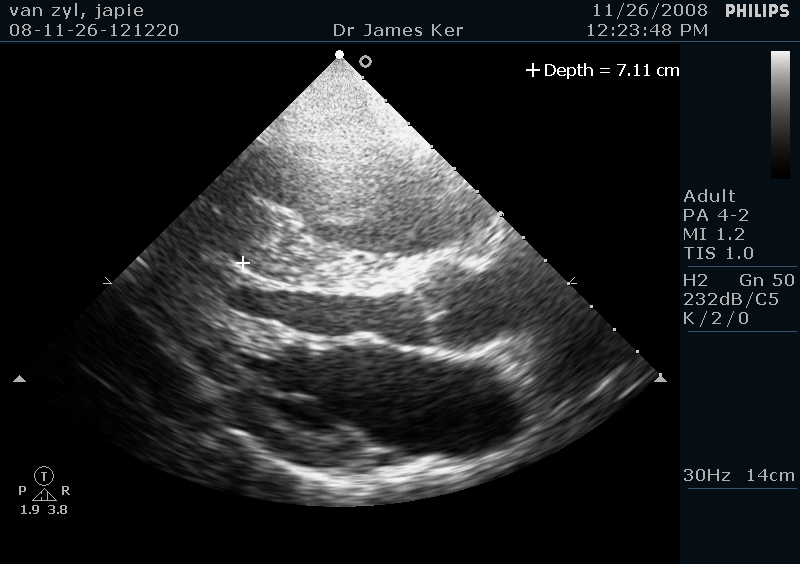

Supplement: Additional file 6 — Septal implantation. Image clearly showing the septal implantation of the muscular tendon. [file 1476-7120-7-13-S6.bmp]
